# Mitochondrial cristae density is increased following high-intensity interval training in men with type 2 diabetes

**DOI:** 10.1007/s00125-026-06694-6

**Published:** 2026-03-08

**Authors:** Martin E. de Almeida, Niels Ørtenblad, Amalie B. Platz, Maria H. Petersen, Kurt Højlund, Joachim Nielsen

**Affiliations:** 1 Department of Sports Science and Clinical Biomechanics, Faculty of Health Sciences, University of Southern Denmark, Odense, Denmark; 2 Steno Diabetes Center Odense, Odense University Hospital, Odense, Denmark; 3 Department of Clinical Research, Faculty of Health Sciences, University of Southern Denmark, Odense, Denmark

**Keywords:** High-intensity interval training, Mitochondria, Mitochondrial cristae density, Skeletal muscle fibres, Transmission electron microscopy, Type 2 diabetes

## Abstract

**Aims/hypothesis:**

Mitochondrial cristae architecture is a key determinant of oxidative capacity in skeletal muscle. While mitochondrial dysfunction is common in type 2 diabetes, it remains unclear whether cristae density is reduced and whether it can be improved by exercise training. We therefore investigated the mitochondrial cristae density in skeletal muscle of individuals with type 2 diabetes compared with glucose-tolerant individuals with obesity and lean individuals, and examined the effect of high-intensity interval training (HIIT).

**Methods:**

In a non-randomised intervention study, the effect of an 8 week supervised HIIT intervention combining rowing and cycling was examined in male participants (aged 40–65 years) with type 2 diabetes (*n*=15), glucose-tolerant individuals with obesity (*n*=15), and lean individuals (*n*=18). Muscle biopsies from the vastus lateralis muscle were analysed using transmission electron microscopy to quantify mitochondrial cristae density (cristae surface area per mitochondrial volume) and to derive cristae surface area per muscle volume, integrating mitochondrial abundance and ultrastructure. To ensure high stereological precision, a minimum of 49 mitochondrial profiles per sample were analysed.

**Results:**

No differences in mitochondrial cristae density were observed between groups at baseline. HIIT induced a ~7% increase in cristae density across all groups, with the most pronounced adaptations in type 2 fibres and in the intermyofibrillar compartment. At baseline, participants with type 2 diabetes exhibited lower cristae surface area per muscle volume compared with lean individuals. Notably, HIIT increased cristae surface area per muscle volume by ~55%, exceeding the magnitude of previously reported increases in mitochondrial volume density.

**Conclusions/interpretation:**

Skeletal muscle mitochondrial cristae density is not different between individuals with type 2 diabetes and glucose-tolerant individuals with obesity and lean individuals, and the capacity for cristae remodelling in response to exercise is not affected by type 2 diabetes. These findings highlight the plasticity of mitochondrial architecture and support HIIT as a potent stimulus for improving muscle oxidative and metabolic health in type 2 diabetes.

**Graphical Abstract:**

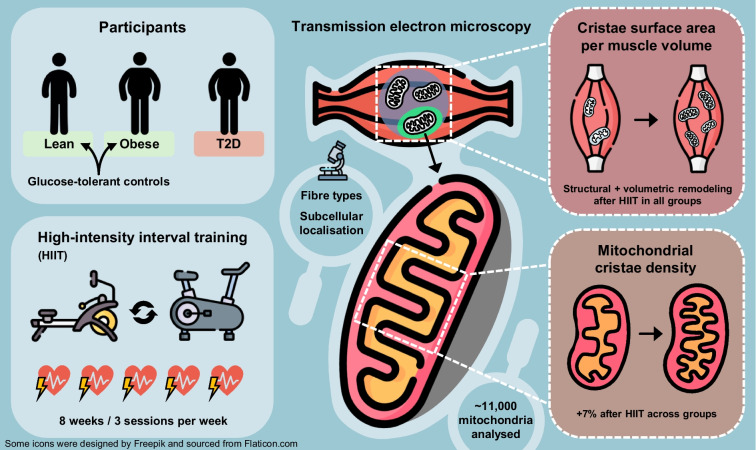

**Supplementary Information:**

The online version contains peer-reviewed but unedited supplementary material available at 10.1007/s00125-026-06694-6.


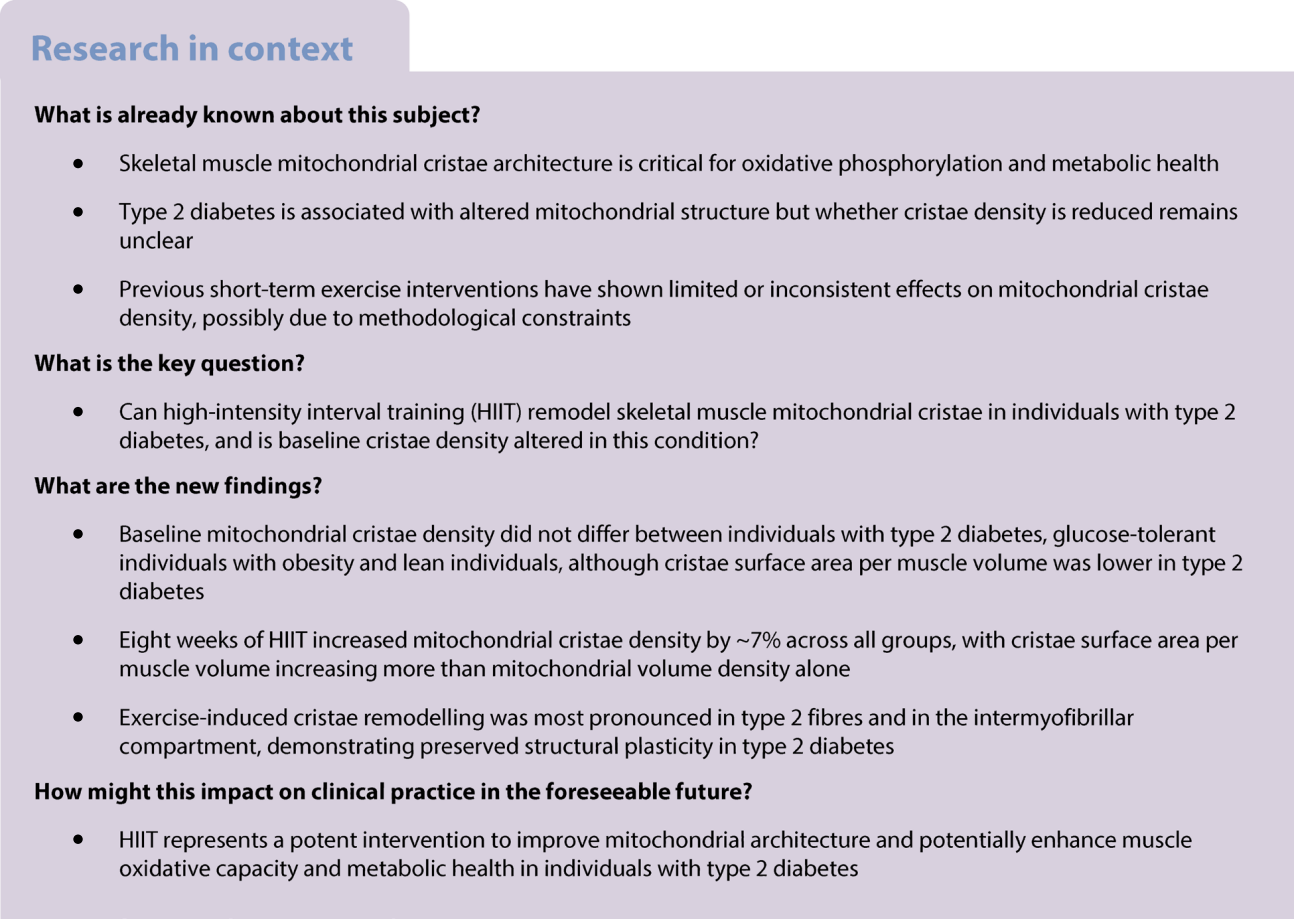


## Introduction

Mitochondria are dynamic organelles essential for cellular energy conversion, metabolic regulation, and signalling [1]. They are enclosed by an outer and inner membrane with distinct protein compositions. The inner membrane forms invaginations into the matrix, termed cristae [2], encompassing the respiratory chain complexes, ATP synthase, the mitochondrial contact site and cristae organising system (MICOS), and other factors that shape cristae architecture and function [3, 4]. Regulation of cristae architecture and density is therefore critical for oxidative phosphorylation.

In skeletal muscle from individuals with type 2 diabetes, the mitochondrial network is frequently fragmented and structurally altered [5–9]; this has been linked to reduced oxidative metabolism [10, 11] and impaired oxidative phosphorylation capacity in most studies [9, 12–17], although some have reported preserved function [18–20]. Whether cristae are affected remains uncertain. At the ultrastructural level, reduced mitochondrial cristae density has been observed in myotubes derived from individuals with type 2 diabetes [21] and altered cristae morphology has been found in mouse models of early diabetes [22], whereas muscle biopsy specimens from individuals with type 2 diabetes showed no difference compared with specimens from weight-matched control individuals [23]. At the molecular level, proteins critical for maintaining cristae architecture, including MICOS components and the cristae-shaping GTPase optic atrophy 1 (OPA1), have been reported to be reduced in abundance in insulin-resistant muscle [24–26]. Notably, OPA1 levels correlate positively with insulin sensitivity [5, 25], suggesting a potential link between cristae organisation and metabolic health, although not all studies have observed lower OPA1 levels in type 2 diabetes compared with weight-matched control individuals [5, 27].

Exercise training is a potent stimulus for mitochondrial remodelling [28, 29]. While intervention studies have generally reported no effect on mitochondrial cristae density following short-term training [23, 30], cross-sectional comparisons demonstrate ~20% higher cristae density in endurance-trained athletes compared with untrained individuals [23], suggesting that the training stimulus in those interventions may have been insufficient. Because cristae surface area density in human skeletal muscle is typically reported within a relatively narrow range (~25–35 µm^2^ µm⁻^3^) [23, 31–33], small but physiologically meaningful differences between populations or following short-term training may be difficult to detect due to both methodological and biological variability inherent to morphometric analyses. This highlights the importance of applying high-precision morphometric approaches in intervention studies to reliably capture potential remodelling of cristae architecture.

To address this limitation, we aimed to investigate, with increased sampling precision, both baseline mitochondrial cristae density and the effect of an 8 week high-intensity interval training (HIIT) intervention in skeletal muscles of individuals with type 2 diabetes, weight-matched individuals with obesity and lean individuals. We hypothesised that the individuals with type 2 diabetes would display lower mitochondrial cristae density at baseline compared with both individuals with obesity and lean individuals, and that HIIT would increase cristae density across all groups.

## Methods

### Ethics

The study was approved by the Regional Committees on Health Research Ethics for Southern Denmark (S-20170142) and the Danish Data Protection Agency (17/31977) and conducted in accordance with the principles outlined in the Declaration of Helsinki. All participants received both oral and written information about the study, including potential risks, before providing written informed consent. Data management complied with the General Data Protection Regulation (GDPR) and was securely handled in REDCap, hosted by Odense Patient Data Explorative Network (OPEN).

### Participants

**Fig. 1:**
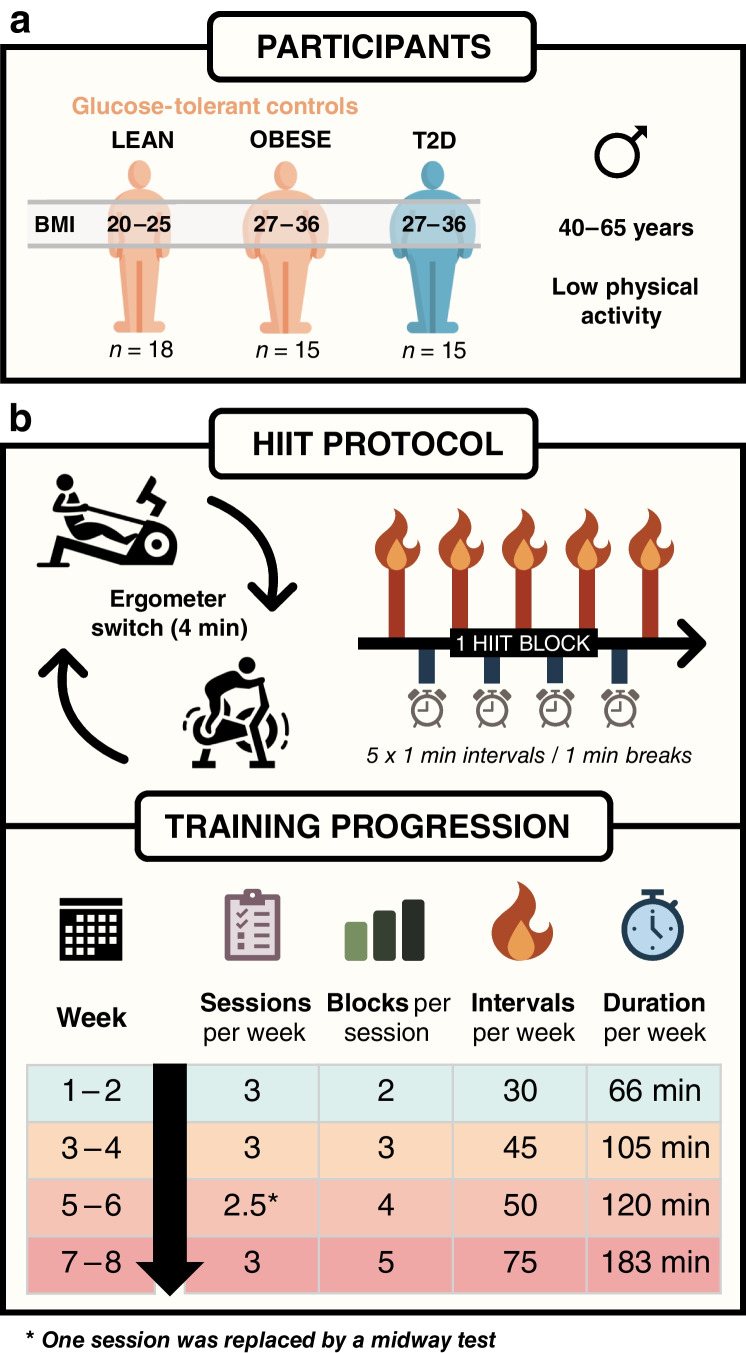
Schematic overview of participants and the 8-week HIIT protocol. (**a**) Three groups were included: lean glucose-tolerant individuals (BMI 20–25 kg/m^2^, *n*=18), glucose-tolerant individuals with obesity (BMI 27–36 kg/m^2^, *n*=15) and individuals with type 2 diabetes (BMI 27–36 kg/m^2^, *n*=15). All participants were men, 40–65 years old, who reported a low physical activity level. (**b**) Participants completed 24 planned sessions combining rowing and cycling on ergometers; one session was replaced by a mid-way test to reassess training intensity. Each session consisted of HIIT blocks of 5×1 min intervals separated by 1 min rest. During a 4 min transition between blocks, participants switched ergometers (rowing–cycling). The number of blocks per session increased biweekly from 2 (weeks 1–2) to 5 (weeks 7–8), resulting in a weekly increase of HIIT intervals from 30 to 75 and total weekly training duration from 66 to 183 min. Some icons were designed by surang and gravisio, sourced from Flaticon.com. T2D, type 2 diabetes

This study is a prespecified secondary analysis of a larger controlled trial, previously described in companion publications [6, 34–36]. Recruitment and intervention took place between January 2018 to December 2019. Fifteen individuals with type 2 diabetes and overweight/obesity (BMI 27–36 kg/m^2^) were matched to 18 lean individuals (BMI 20–25 kg/m^2^) and 15 non-diabetic individuals with overweight/obesity (BMI 27–36 kg/m^2^) (Fig. 1a). Clinical and biochemical characteristics of the three groups are presented in Table 1. Eligible participants were men aged 40–65 years who were classified as sedentary according to the International Physical Activity Questionnaire – Short Form (IPAQ–SF), reporting <2 h of moderate-intensity activities per week. All participants had a normal resting ECG and normal blood screening for renal, hepatic and haematological function. Participants with type 2 diabetes were GAD65 antibody-negative, without micro- or macrovascular complications (except one with mild retinopathy), and treated with metformin (*n*=14), dipeptidyl peptidase-4 (DPP-4) inhibitors (*n*=4), or sulfonylureas (*n*=1), either as monotherapy (*n*=11) or in combination (*n*=4). They also received cholesterol-lowering (*n*=11) and antihypertensive (*n*=12) medications. Participants in the control groups were glucose-tolerant based on a 2 h 75 g OGTT, drug-naive and without a family history of diabetes. Sex was self-reported in accordance with their Danish civil registration number. Data on race and ethnicity were not collected, as these are considered social constructs without biological relevance to the aims of this study.

**Table 1:** Clinical and biochemical characteristics at baseline

Characteristic	Lean (*n*=15)	Obese (*n*=14)	T2D (*n*=14)
Age (years)^a^	56.2±6.2	54.0±7.1	54.6±6.2
Duration of diabetes (years)			4.6±2.9
Body weight (kg)	81.2±6.7	98.9±10.7***	104.2±14.3***
BMI (kg/m^2^)	24.3±1.2	30.6±2.7***	31.4±3.1***
Fat mass (kg)	20.9±4.0	31.4±7.3***	35.7±8.3***
Fat-free mass (kg)	58.4±4.5	65.1±5.1**	64.9±6.8**
V̇O_2max_ (ml/kg/min)	37.3±6.4	33.5±7.3	25.6±3.4***^†††^
Maximal fat oxidation (mg/min)^a^	215±116	184±128	145±103
HbA_1c_ (mmol/mol)	35.1±2.8	34.8±3.4	51.5±11.6***^†††^
HbA_1c_ (%)	5.4±0.3	5.3±0.3	6.9±1.1***^†††^
Fasting plasma glucose (mmol/l)	5.2±0.4	5.6±0.5	9.4±2.8***^†††^
Serum insulin (pmol/l)	59±32	71±34	121±77**^†^
Plasma triacylglycerol (mmol/l)	1.41±0.65	1.48±0.61	2.49±1.59*^†^
LDL-cholesterol (mmol/l)	3.21±0.71	3.63±0.53	2.95±0.64^††^
HDL-cholesterol (mmol/l)	1.23±0.34	1.25±0.18	1.02±0.27*^†^
GDR, clamp (mg min^−1^ m^−2^)	368±132	353±103	198±82***^†††^

Values are presented as means ± SD; data are adapted from [6, 34]

Group comparisons were conducted using linear regression analysis

^a^Main effect of group: NS

^*^p<0.05, **p<0.01, ***p<0.001 vs lean

^†^*p*<0.05, ^††^*p*<0.01, ^†††^*p*<0.001 vs obese

GDR, glucose disposal rate; T2D, type 2 diabetes

### Experimental protocol

Participants completed an 8 week HIIT intervention and visited the laboratory on two experimental days (≥48 h apart) both before and after the intervention. On each visit, they arrived at 07:30 hours in a fasted state (≥12 h), having abstained from alcohol and caffeine (≥24 h) and from strenuous physical activity (≥48 h). Participants with type 2 diabetes were instructed to withhold all medications (glucose-lowering, lipid-lowering and antihypertensive) 1 week prior to each visit. Participants were instructed to maintain their habitual dietary intake throughout the intervention. All procedures were supervised by personnel experienced in physiological and metabolic testing.

On the first experimental day, whole-body mass and composition were assessed by dual-energy x-ray absorptiometry (DXA) (Prodigy Advance; GE Healthcare, WI, USA). Participants then performed a graded exercise test on a cycle ergometer (SRM Ergometer System; Jülich, Germany) consisting of 4 min stages starting at 40–60 W, with 20 W increments until respiratory exchange ratio (RER) reached 1.0, followed by 1 min 20 W increments to exhaustion. This test was used to determine substrate oxidation rates and cardiorespiratory fitness ($\dot{V}{\mathrm{O}}_{2\mathrm{max}}$; calculated as the highest 30 s average). Maximal effort was considered valid if at least two of the following three criteria were met: $\dot{V}{\mathrm{O}}_{2}$ plateau <2.1 ml kg^−1^ min^−1^; RER >1.1; or post-exercise lactate >8 mmol/l. Two hours before testing, participants consumed a standardised mixed breakfast (25 kJ/kg body mass [6 kcal/kg]; 64% carbohydrate, 22% fat, 14% protein). Full methodological details on the graded exercise test and calculation of maximal fat oxidation, including previously reported training-induced changes in $\dot{V}{\mathrm{O}}_{2\mathrm{max}}$ and substrate oxidation rates, have been described elsewhere [6, 34]. After the intervention, this first day was conducted ~60 h after the final HIIT session.

On the second experimental day, a resting skeletal muscle biopsy was obtained, followed by a 3 h hyperinsulinaemic–euglycaemic clamp to assess insulin sensitivity. Clamp methodology, isotope infusion protocols and biochemical analyses of plasma glucose, HbA_1c_, C-peptide, serum insulin and lipids have been detailed in a separate prior publication [34]. Post-intervention, this experimental day was scheduled 48 h after the first day (including $\dot{V}{\mathrm{O}}_{2\mathrm{max}}$ test) and 5 days after the final HIIT session.

### Training intervention

The 8 week intervention (Fig. 1b) comprised of three weekly HIIT sessions combining rowing (Concept2 Model E; VT, USA) and cycling (Wattbike Pro/Trainer; Nottingham, UK) ergometry, totalling 24 sessions, including one mid-way test to reassess training intensity. Participants across all groups trained together in groups of up to ten individuals, with most sessions (>95%) conducted in the afternoon. Each session began with a 10 min warm-up, followed by HIIT blocks consisting of 5×1 min intervals interspersed with 1 min recovery (passive rest or low-intensity active recovery). A 4 min rest period was provided between blocks to switch between rowing and cycling, with the starting modality alternating between sessions. Training volume increased biweekly, from two blocks per session in weeks 1–2 to five blocks in weeks 7–8. Cycling intervals were performed at 100–110% of maximal cycling capacity (MCC), with similar heart rates achieved during rowing and cycling (86–88% of maximum). Rowing ergometers were calibrated with a drag factor of 105–110, and participants received real-time feedback to ensure supramaximal effort. Motivation was supported by group dynamics, supervisor encouragement, music, and live heart rate monitoring (Polar H7/Team, Kempele, Finland). Detailed training data are reported in [6].

### Muscle biopsies

All skeletal muscle biopsies were collected from the vastus lateralis muscle by the same experienced physician to minimise variation in sampling depth and location. Following local anaesthesia (5 ml of 2% wt/vol. lidocaine), a small incision was made through the skin and fascia, and the biopsy was obtained using a modified Bergström needle with suction. Samples were immediately cleared of blood and connective tissue, then divided for various analyses, including a ~1 mm^3^ portion prepared for transmission electron microscopy (TEM).

Due to a fixation error, pretraining biopsies were unavailable for five participants (three lean individuals, one individual with obesity and one with type 2 diabetes), who were excluded from the analyses. In addition, four participants did not complete the intervention: one lean individual and two individuals with type 2 diabetes withdrew due to time constraints, and one lean individual did not initiate training due to a knee injury. However, their baseline characteristics were retained in the present study. To increase tissue availability for mitochondrial analyses, additional pretraining biopsies were included from several of the participants (11 lean individuals, 12 individuals with obesity and 12 with type 2 diabetes). These biopsies were a part of a separate acute exercise study [35] conducted on the same participants as enrolled in the present study and performed within the same pre-intervention timeframe. All samples were collected ≥48 h after experimental day 2 and prior to initiation of the HIIT intervention, ensuring that all pretraining samples represent the same physiological state. The final analytical sample was comprised of individuals with type 2 diabetes (pre *n*=14; post *n*=12); individuals with obesity (pre/post *n*=14), and lean individuals (pre *n*=15; post *n*=13). The baseline characteristics reported in Table 1 are based on available participants with valid pretraining biopsies.

### TEM

**Fig. 2:**
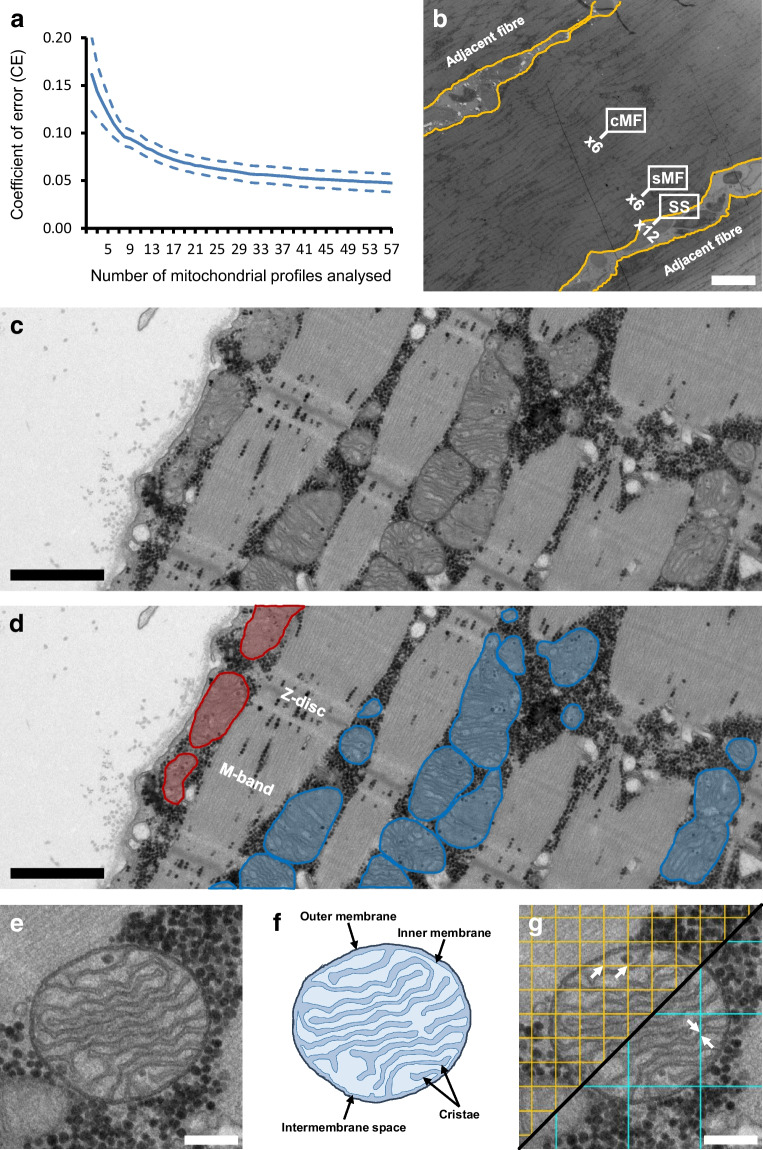
TEM and stereological assessment of skeletal muscle mitochondria. (**a**) To ensure high precision of cristae density estimates, ≥49 mitochondrial profiles per sample were analysed, yielding an _est_CE <5%. The continuous line indicates the mean _est_CE, while the dashed lines represent the 95% CI of the _est_CE. (**b**) Micrographs were acquired from ten longitudinally oriented fibres per biopsy, with 24 images per fibre: 12 from the subsarcolemmal (SS) region; six from the superficial miofibrillar (sMF) region; and six from the central (cMF) myofibrillar region. (**c**) Representative TEM image of a skeletal muscle fibre. (**d**) The same image with subsarcolemmal mitochondria highlighted in red and intermyofibrillar mitochondria highlighted in blue. (**e**) Example of a mitochondrion of acceptable quality, showing clearly visible cristae structure with minimal or no missing traces. (**f**) Schematic illustration of mitochondrial morphology, including outer membrane, inner membrane with cristae, and intermembrane space. (**g**) Grids were overlaid to quantify intersections of inner membrane folds (teal lines) for cristae surface area and mitochondrial hits (orange lines) for volume; the ratio of cristae surface area to mitochondrial volume was used to calculate cristae density. Arrows indicate example points hitting the mitochondrion and intersections of cristae membranes. Scale bars, 10 µm (**b**), 1 µm (**c**, **d**), 200 nm (**e**, **g**)

Muscle biopsy specimens were prepared for TEM as described previously [37]. Briefly, samples were fixed in 2.5% glutaraldehyde in a 0.1 mol/l sodium cacodylate buffer (pH 7.3) for 24 h at 5°C and subsequently washed four times in the same buffer. Post-fixation was carried out in a solution of 1% osmium tetroxide (OsO_4_) and 1.5% potassium ferrocyanide [K_4_Fe(CN)_6_] in 0.1 mol/l sodium cacodylate buffer for 120 min at 4°C. Following post-fixation, the specimens were rinsed twice in double-distilled water, dehydrated through a graded ethanol series, and infiltrated with graded mixtures of propylene oxide and Epon at room temperature. The following day, samples were embedded in 100% Epon resin and polymerised at 60°C for 48 h. Ultrathin sections (60 nm) were cut using an Ultracut UC7 ultramicrotome (Leica, Wetzlar, Germany) at three depths separated by 150 µm to maximise fibre sampling. Sections were contrasted with uranyl acetate and lead citrate before examination in a pre-calibrated JEM-1400plus transmission electron microscope (JEOL, Tokyo, Japan) equipped with a CCD camera (Quemesa, Olympus, Tokyo, Japan). Electron micrographs were acquired from ten longitudinally oriented muscle fibres per biopsy. For each fibre, 24 images were captured at ×6000 magnification in a randomised systematic order: 12 from the subsarcolemmal region; and six from the superficial and central region of the myofibrillar space (Fig. 2b).

Muscle fibres were classified as type 1 or type 2 based on their distinct Z-disc width, which correlates with myofibrillar ATPase staining patterns in light microscopy [38, 39]. Fibres with the thickest Z-discs were classified as type 1, and those with the thinnest as type 2. Three fibres of each type were selected per biopsy for point-counting analysis, while intermediate fibres were excluded. The median (IQR) Z-disc width was 83 (78–89) nm for type 1 fibres and 65 (62–69) nm for type 2 fibres.

### Mitochondrial volume density

Mitochondrial volume density for the three groups was previously determined [6], and included data on stereological precision and inter-investigator reproducibility. As described by Hoppeler et al [32], total mitochondrial volume density in skeletal muscle encompasses both subsarcolemmal mitochondria, located between the sarcolemma and the outermost myofibrils, and intermyofibrillar mitochondria, which are positioned between the myofibrils, typically near the I-band and adjacent to the Z-disc (Fig. 2c, d).

Two subcellular compartments were defined for stereological estimation: (1) the intermyofibrillar space; and (2) the subsarcolemmal space. The volume density of intermyofibrillar mitochondria was expressed relative to the myofibrillar space and estimated by counting points in both the superficial and central regions of the myofibrillar compartments. Given that muscle fibres are assumed to be cylindrical with a diameter of 80 µm [40], and that the superficial region occupies approximately three times the volume of the central region, counts from the superficial region were weighted three times more heavily. In contrast, the volume density of subsarcolemmal mitochondria was expressed relative to the surface area of the muscle fibre, based on direct measurements of fibre length in the subsarcolemmal region (i.e. visible myofibrils parallel to the sarcolemma). Fibre surface area was then calculated as the measured fibre length multiplied by the thickness of the ultrathin sections (60 nm).

Volume density (V_v_) was estimated using a 350×350 point grid (442 test points per micrograph), applying the stereological principle that volume density can be approximated from point density (P_p_) as V_v_=A_a_=P_p_, where A_a_ is the area density [41]. Since intermyofibrillar mitochondria were expressed per myofibrillar volume and subsarcolemmal mitochondria per surface area, total volume densities were computed by normalising the subsarcolemmal volume densities to the muscle fibre volume. This was done by dividing the subsarcolemmal values by a factor of 20, based on the geometric assumption (V_b_=r×0.5A) that the volume beneath (V_b_) 1 mm^2^ of surface area (A) in a fibre with 40 µm radius (r) is 20 mm^3^. All analyses were performed in iTEM (Olympus, Tokyo, Japan).

### Mitochondrial cristae density

Mitochondrial cristae density, defined as cristae surface area per mitochondrial volume, was quantified on the same electron micrographs used in the previous point-counting analysis. Mitochondrial profiles were included if they met quality criteria, defined by clear visibility and minimal disruption or loss of the inner mitochondrial membrane (Fig. 2e, f). If only part of a mitochondrial profile fulfilled these criteria, then that specific segment was included in the analysis. Initially, all mitochondrial profiles present in each micrograph were analysed. However, due to the high image quality and the substantial time required, this approach proved impractical. Therefore, the strategy was revised to include one mitochondrial profile per micrograph. Selection was conducted systematically by visually scanning each image from the top-left to the bottom-right corner and including the first profile that met the predefined quality criteria. This adjustment, implemented after the completion of the first of 14 folders, was made to minimise selection bias. In total, 10,851 (whole or partial) mitochondrial profiles were analysed (Pre: 6087; Post: 4764), with a median (IQR) of 134 (72–218) and 117 (86–143) profiles per participant at Pre and Post, respectively.

Quantification of mitochondrial cristae density (SV) followed Weibel’s stereological principles [41], using the standard formula SV=2×I_dbl_×(P_mi_ ×*k*×*d*)^−1^, where I_dbl_ is the number of intersections between the inner membrane and test lines, P_mi_ is the number of test points hitting the mitochondrial profile (Fig. 2g), and $k$ and $d$ are constants defined by the test system. A grid size of 270×270 nm and 90×90 nm was used to measure I_dbl_ and P_mi_, respectively. All analyses were conducted in ImageJ (version 1.54d; National Institute of Health, MD, USA). Finally, to estimate the cristae surface area per muscle volume, mitochondrial cristae density was multiplied by mitochondrial volume density.

Previous assessments of inter-mitochondrial profile variation [23, 31] have shown that a minimum of eight mitochondrial profiles per sample are necessary to obtain an estimated coefficient of error (_est_CE) of 0.10. However, as the expected effect size of training on mitochondrial cristae density is likely below 10% [23], we aimed for an _est_CE of 0.05, corresponding to a minimum of 49 mitochondrial profiles per estimate (Fig. 2a). This stringent criterion, applied across fibre types (mixed fibres), led to the exclusion of ten pre-biopsies and six post-biopsies across the three groups. The number of exclusions increased further when stratifying by fibre type and subcellular location. For transparency, results based on the less-stringent threshold of only eight profiles (and thus fewer excluded samples) are also presented (electronic supplementary material [ESM] Figs 1, 2). Two investigators examined the eligible mitochondrial profiles in randomised order. They were blinded to group, time point and muscle fibre type, with micrographs evenly distributed across all factors. Cross-validation of their inter-investigator scoring revealed a CV of 4.7% for the cristae density estimates based on 49 mitochondrial profiles.

### Statistics

In alignment with the recommendations by Ho et al [42], this study employed estimation statistics to evaluate training-induced effect size along with their associated 95% CI. Effect size was visualised using estimation plots, including paired comparisons across time, stratified by group, fibre type and subcellular location. The magnitude of the effects was interpreted as small (0.20–0.49), moderate (0.50–0.79), or large (≥0.80), based on Cohen’s *d* [43]. Changes (∆) were calculated for all participants, and ∆-∆ comparisons were performed to assess differences in responses between the three groups. To complement the estimation approach and account for missing values, a linear mixed-effects model was also applied to test interactions and main effects. Participants were treated as random effects, and time, group and fibre type as fixed effects. Normality was evaluated using the Shapiro–Wilk test, Skewness–Kurtosis test and Q–Q plots. Homoscedasticity was assessed using residual plots. Variables with skewed distributions were transformed prior to analysis. Main effects and interactions were evaluated by the Wald χ^2^ test. Group differences in participant characteristics were assessed using linear regression. Pearson’s correlation coefficients were computed between mitochondrial markers and metabolic variables, as visual inspection indicated a linear relationship between the variables (approximated as *y*=*a*×*x*+*b*). Statistical analyses were conducted using StataBE (version 18.5; StataCorp, TX, USA), while figures were created in Excel (Microsoft, WA, USA). Data are presented as means ± 95% CI or medians with IQR, unless otherwise stated. The threshold for statistical significance was set at *p*<0.05.

## Results

As previously reported [6, 34], insulin-stimulated glucose disposal rate at baseline was markedly lower in the participants with type 2 diabetes compared with those with obesity and lean individuals (Table 1). HIIT improved glucose disposal rate by ~30–40% across groups and was accompanied by increases in $\dot{V}{\mathrm{O}}_{2\mathrm{max}}$ (~8–15%) and maximal fat oxidation (~40–60%), reductions in total fat mass (~1.6–2.3 kg), and increases in lean body mass (~0.6–1.5 kg), with no between-group differences.

### Mitochondrial cristae density across groups and in response to HIIT

**Fig. 3:**
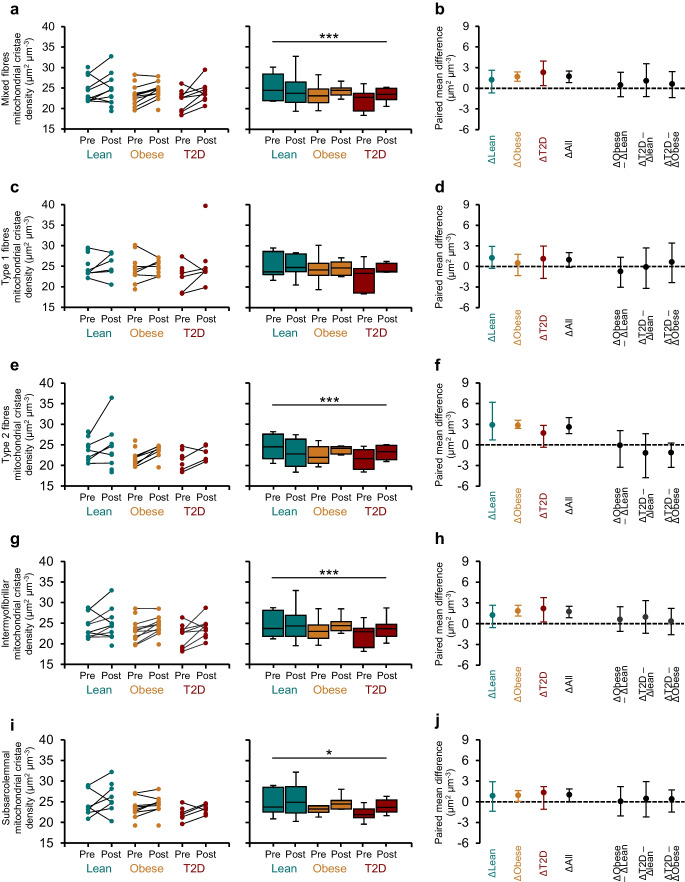
Effect of HIIT on mitochondrial cristae density in skeletal muscle fibres and subcellular regions. Cristae density is shown for mixed (**a**, **b**), type 1 (**c**, **d**) and type 2 fibres (**e**, **f**), and intermyofibrillar (**g**, **h**) and subsarcolemmal regions (**i**, **j**). Individual pre- and post-HIIT values (slope graphs) are shown alongside group-wise distributions (boxplots with medians and IQRs), and corresponding paired mean differences and ∆-∆ comparisons. Mixed-effects model results (main effect of group, main effect of time, and the group × time interaction) were as follows: mixed fibres, group *p*=0.505, time *p*<0.001, interaction *p*=0.110; type 1 fibres, group *p*=0.804, time *p*=0.071, interaction *p*=0.648; type 2 fibres, group *p*=0.714, time *p*<0.0001, interaction *p*=0.220; intermyofibrillar region, group *p*=0.376, time *p*<0.001, interaction *p*=0.581; and subsarcolemmal region, group *p*=0.165, time *p*=0.017, interaction *p*=0.664. *p<0.05, ***p<0.001. Data from participants were included if ≥49 mitochondrial profiles were analysed (_est_CE <5%). Individual data and boxplots: lean, *n*=9–11 pre, *n*=7–12 post; obese, *n*=11–12 pre, *n*=8–12 post; type 2 diabetes, *n*=7–10 pre, *n*=6–9 post. Paired mean differences: lean, *n*=6–9; obese, *n*=6–10; type 2 diabetes, *n*=4–8. T2D, type 2 diabetes

No differences in mitochondrial cristae density were observed between groups at either baseline or post-intervention (Fig. 3a, c, e, g, i). At baseline, mitochondrial cristae density was ~6% higher in type 1 fibres than in type 2 fibres (main effect of fibre type *p*<0.0001; Fig. 3c, e) but this difference was no longer evident after HIIT.

HIIT increased mitochondrial cristae density by 1.7 µm^2^ µm^−3^ (paired mean difference, Fig. 3b), corresponding to a ~7% relative increase from baseline and a moderate effect size (Cohen’s *d* = 0.65). This change occurred independently of type 2 diabetes or obesity. The effect was primarily driven by adaptations in type 2 fibres, which showed a ~12% increase (Cohen’s *d* = 0.85; Fig. 3f), whereas type 1 fibres exhibited smaller changes (Cohen’s *d* = 0.35; Fig. 3d). Among subcellular compartments, the strongest effect was observed in the intermyofibrillar compartment (Cohen’s *d* = 0.64; Fig. 3h), with more modest adaptations in subsarcolemmal mitochondria (Cohen’s *d* = 0.41; Fig. 3j).

No between-group differences were detected in the ∆-∆ changes from pre- to post-HIIT (Fig. 3b, d, f, h, j). However, based on the observed variation within the 95% CI, it cannot be excluded that individuals with type 2 diabetes or obesity may have experienced greater training-induced improvements in cristae density, potentially starting from a lower pretraining level than lean individuals.

### Cristae surface area per muscle volume across groups and in response to HIIT

We previously demonstrated that mitochondrial volume density is largely preserved in individuals with type 2 diabetes, with no apparent differences in either the intermyofibrillar or subsarcolemmal compartments when compared with individuals with obesity and lean individuals [6]. In that study, HIIT led to substantial increases in mitochondrial volume density across all three groups (~20–40% in the intermyofibrillar region and ~30–50% in the subsarcolemmal region). To examine whether mitochondrial remodelling extends beyond volumetric expansion, we estimated cristae surface area per muscle volume. This composite metric captures both mitochondrial abundance and internal structure.

**Fig. 4:**
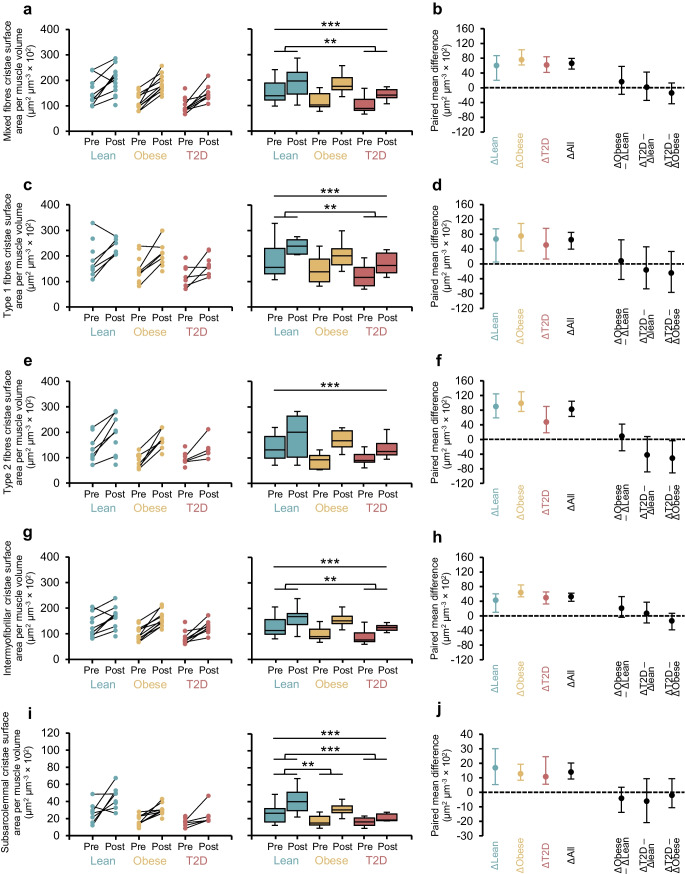
Effect of HIIT on mitochondrial cristae surface area per muscle volume in skeletal muscle fibres and subcellular regions. Cristae surface area per muscle volume is shown in mixed fibres (**a**, **b**), type 1 fibres (**c**, **d**) and type 2 fibres (**e**, **f**), and intermyofibrillar (**g**, **h**) and subsarcolemmal regions (**i**, **j**). Panel layout and presentation of individual data, boxplots, paired mean differences, and ∆-∆ comparisons are as for Fig. 3. Mixed-effects model results (main effect of group, main effect of time, and the group × time interaction) were as follows: mixed fibres, group *p*=0.012, time *p*<0.0001, interaction *p*=0.410; type 1 fibres, group *p*=0.008, time *p*<0.0001, interaction *p*=0.912; type 2 fibres, group *p*=0.098, time *p*<0.0001, interaction *p*=0.122; intermyofibrillar region, group *p*=0.014, time *p*<0.0001, interaction *p*=0.217; and subsarcolemmal region, group *p*<0.0001, time *p*<0.0001, interaction *p*=0.526. **p<0.01, ***p<0.001. Data from participants were included if ≥49 mitochondrial profiles were analysed (_est_CE <5%). Individual data and boxplots: lean, *n*=9–11 pre, *n*=7–12 post; obese, *n*=11–12 pre, *n*=8–12 post; type 2 diabetes, *n*=7–10 pre, *n*=6–9 post. Paired mean differences: lean, *n*=6–9; obese, *n*=6–10; type 2 diabetes, *n*=4–8. T2D, type 2 diabetes

At baseline, cristae surface area per muscle volume was higher in type 1 fibres than in type 2 fibres, and this fibre-type difference persisted after training (main effect of fibre type, both *p*<0.001; Fig. 4c, e). While mitochondrial volume density alone did not reveal clear group differences [6], the combined measure indicated higher values in lean individuals than in those with type 2 diabetes, particularly in type 1 fibres (Fig. 4c). This suggests that integrating volumetric and structural features may improve sensitivity to subtle group differences, although the observed pattern appeared primarily driven by changes in mitochondrial volume density.

Given the increase in mitochondrial cristae density following HIIT, the composite measure of cristae surface area per muscle volume increased to a greater extent than mitochondrial volume density alone (~55% vs ~20–50% reported previously), with increases across all groups of ~55% in mixed fibres, ~45–80% in type 1 and type 2 fibres, and ~50–70% in the intermyofibrillar and subsarcolemmal compartments (all Cohen’s *d* >1; Fig. 4b, d, f, h, j).

No between-group differences in ∆-∆ change of cristae surface area per muscle volume were observed for type 1 fibres (Fig. 4d) or for mitochondria in the intermyofibrillar and subsarcolemmal compartments (Fig. 4h, j). In type 2 fibres, individuals with obesity initially appeared to display a greater training-induced increase compared with individuals with type 2 diabetes (Fig. 4f). However, this effect was not confirmed in supplemental analyses using a more conservative effect size threshold (_est_CE <0.10; ESM Fig. 2f) and should therefore be interpreted with caution, particularly given the small sample size in the type 2 diabetes group (*n*=4; Fig. 4f).

**Fig. 5:**
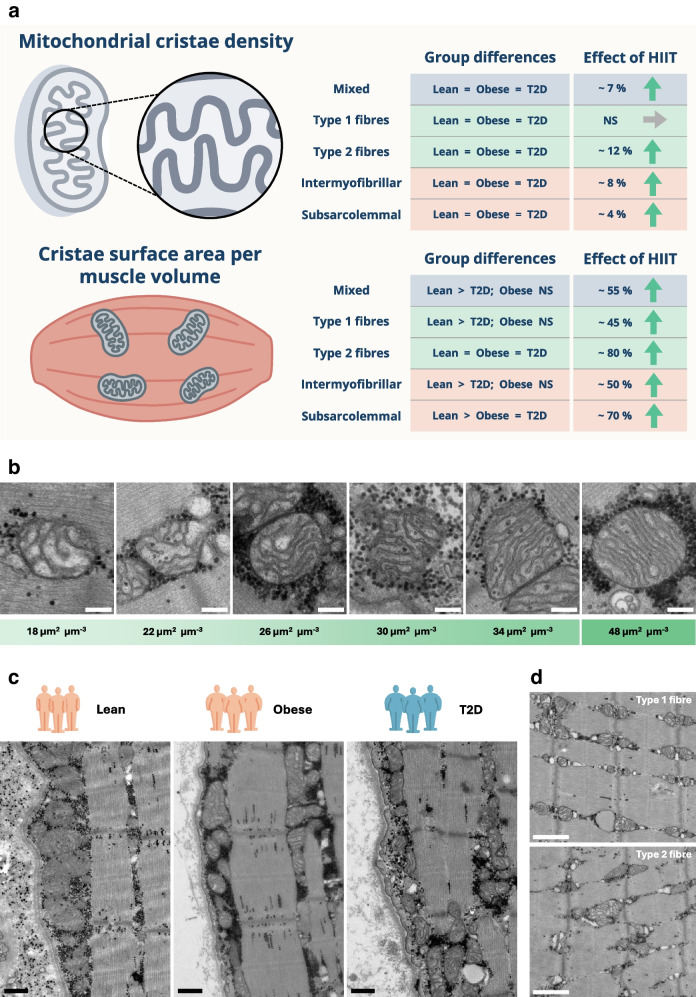
Mitochondrial cristae density and cristae surface area per muscle volume in skeletal muscle. (**a**) Summary of group differences and training-induced changes in mitochondrial cristae (cristae surface area per mitochondrial volume and cristae surface area per muscle volume). Values are shown for mixed fibres, type 1 fibres and type 2 fibres, and for intermyofibrillar and subsarcolemmal compartments. Arrows indicate the direction and approximate magnitude of percentage change (paired mean differences) following HIIT. (**b**) Representative TEM images of individual mitochondria illustrating typical cristae densities spanning 18–34 µm^2^ µm⁻^3^, together with a rare example of a mitochondrion with high cristae density (48 µm^2^ µm⁻^3^). (**c**, **d**) Representative TEM images illustrating mitochondrial ultrastructure across groups (**c**) and fibre types (**d**). Scale bar, 200 nm (**b**), 500 nm (**c**), 1 μm (**d**). Some icons were designed by surang and sourced from Flaticon.com. T2D, type 2 diabetes

The findings on mitochondrial cristae density and cristae surface area per muscle volume are summarised in Fig. 5a, illustrating group differences and HIIT-induced changes across fibre types and subcellular compartments. Representative TEM images illustrating variation in cristae density are shown in Fig. 5b, while Fig. 5c, d provides examples of mitochondrial ultrastructure across groups and fibre types, highlighting the range of volumetric and structural features.

### Correlations between mitochondrial metrics and aerobic capacity

**Fig. 6:**
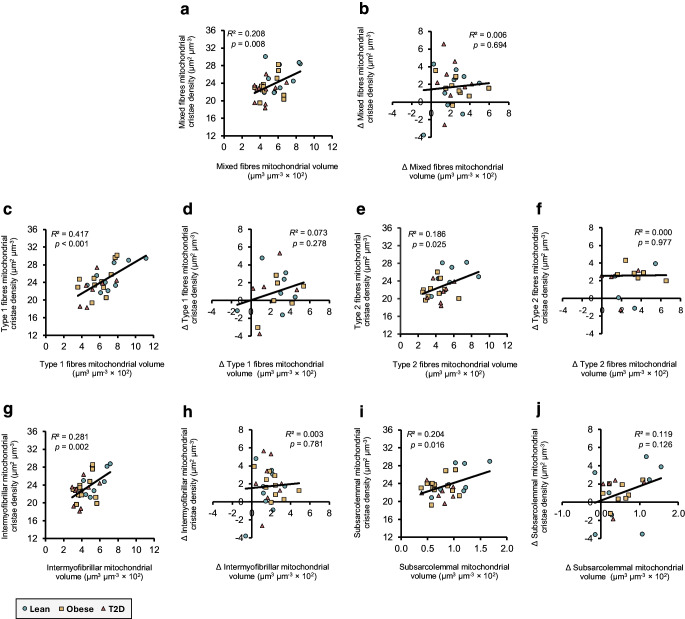
Correlations between mitochondrial volume and cristae density in skeletal muscle fibres and subcellular regions. Correlations at baseline and for delta (∆) change (post–pre) in mixed fibres (**a**, **b**), type 1 fibres (**c**, **d**), type 2 fibres (**e**, **f**) and intermyofibrillar (**g**, **h**) and subsarcolemmal regions (**i**, **j**). Lean (teal circles), baseline *n*=9–11, ∆ *n*=6–9; obese (yellow squares), baseline *n*=11–12, ∆ *n*=6–10); and type 2 diabetes (red triangles), baseline *n*=7–10, ∆ *n*=4–8. Black lines represent the best linear fit including all participants. Coefficient of determination (*R*^2^) and *p* values are reported. T2D, type 2 diabetes

To increase statistical power and focus on overall associations, the three groups were combined for this analysis, and group-specific effects were not examined. Taken together, the observed training-induced changes in mitochondrial structure prompted us to explore associations both among mitochondrial measures themselves and with key measures of aerobic capacity. At baseline, mitochondrial cristae density correlated positively with the mitochondrial volume density across all fibres and subcellular regions (Fig. 6a, c, e, g, i). However, the HIIT-induced increases in cristae density did not correlate with the increases in mitochondrial volume density (Fig. 6b, d, f, h, j).

**Fig. 7:**
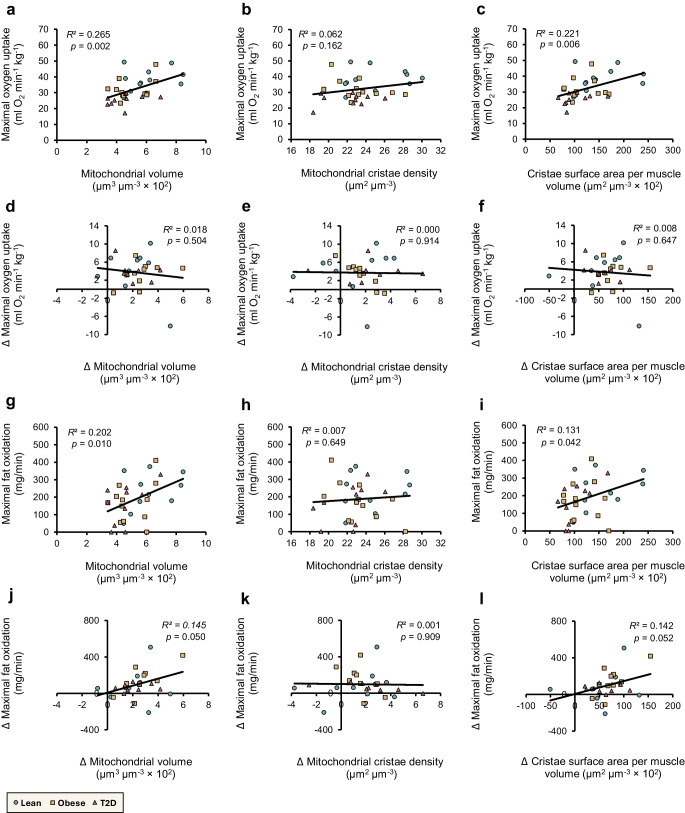
Correlations between mitochondrial morphology and maximal oxygen uptake or fat oxidation. Correlations between mitochondrial volume density, cristae density, and cristae surface area per muscle volume vs maximal oxygen uptake ($\dot{V}{\mathrm{O}}_{2\mathrm{max}}$) (**a**–**f**) and maximal fat oxidation (**g**–**l**), shown at baseline (**a**–**c**, **g**–**i**) and as ∆ change (post–pre) (**d**–**f**, **j**–**l**). Lean (teal circles), baseline *n*=11, ∆ *n*=9; obese (yellow squares), baseline *n*=12, ∆ *n*=10; type 2 diabetes (red triangles), baseline *n*=10, ∆ *n*=8. Black lines represent the best linear fit including all participants. Coefficient of determination (*R*^2^) and *p* values are reported. T2D, type 2 diabetes

At baseline, both maximal oxygen uptake ($\dot{V}{\mathrm{O}}_{2\mathrm{max}}$) and maximal fat oxidation correlated positively with mitochondrial volume density (Fig. 7a, g) and cristae surface area per muscle volume (Fig. 7c, i) but not with cristae density alone (Fig. 7b, h). When examining the shifts in mitochondrial features relative to aerobic metrics, no correlations were found for $\dot{V}{\mathrm{O}}_{2\mathrm{max}}$ (Fig. 7d–f). However, the positive correlations between changes in maximal fat oxidation and changes in mitochondrial volume density (*p*=0.05; Fig. 7j) as well as changes in cristae surface area per muscle volume (*p*=0.052; Fig. 7l) approached statistical significance, while no association was observed with changes in cristae density (Fig. 7k).

## Discussion

Mitochondria play a central role in skeletal muscle energy metabolism, and their structural organisation is a key determinant of oxidative capacity. The ultrastructure of mitochondria, particularly the invaginations of the inner membrane known as cristae, has traditionally been considered relatively invariant in skeletal muscle, regardless of diabetes status or exercise training [33, 44–47]. Here, we distinguish between mitochondrial cristae density, an ultrastructural property defined as cristae surface area per mitochondrial volume, and muscular cristae density, which additionally integrates mitochondrial volume density to reflect the total cristae surface area per muscle volume.

In this study, based on nearly 11,000 mitochondrial profiles imaged by TEM, we demonstrate that mitochondrial cristae density is responsive to HIIT and that this plasticity is preserved in skeletal muscle of individuals with type 2 diabetes. Although mitochondrial cristae density did not differ between individuals with type 2 diabetes, glucose-tolerant individuals with obesity, and lean individuals, cristae surface area per muscle volume was lower in individuals with type 2 diabetes than in lean individuals. Importantly, HIIT increased cristae surface area per muscle volume beyond the previously reported increases in mitochondrial volume density [6], reflecting combined structural and volumetric remodelling.

Our findings of preserved mitochondrial cristae density in individuals with type 2 diabetes contrasts with previous reports of reduced cristae density [21] and disturbed circadian regulation of inner membrane gene expression [25] in myotubes from donors with type 2 diabetes. However, Rajab et al [22] found dysregulated mitochondrial morphology and altered respiratory chain activity in a mouse model of early diabetes despite preserved cristae density. Similarly, we previously observed preserved mitochondrial cristae density in another cohort of individuals with type 2 diabetes [23]. Collectively, these findings underscore the heterogeneity of mitochondrial alterations in diabetes and highlight the unresolved question of whether structural remodelling is impaired in this condition.

Although mitochondrial cristae density was preserved in our study, an integrated measure of cristae surface area per muscle volume revealed higher values in lean individuals than in individuals with type 2 diabetes, particularly for type 1 fibres, with no differences between individuals with type 2 diabetes and individuals with obesity. This pattern appeared to be largely driven by lower mitochondrial volume density in type 2 diabetes, underscoring the importance of incorporating volumetric context to uncover subtle structural differences. Notably, the absence of differences between individuals with type 2 diabetes and individuals with obesity suggests that the underlying mechanism is related to insulin resistance rather than obesity per se. Indeed, individuals with type 2 diabetes display dysregulation of lipid metabolism [6, 10, 14, 16] and mitochondrial dynamics [48] compared with weight-matched individuals without diabetes, supporting this interpretation. In line with this, studies of diabetic myocardium exposed to hypoxia have shown that a composite index of mitochondrial volume, cristae density and ATPase particle abundance provides a more sensitive marker of impaired ATP-generating capacity than single ultrastructural parameters [49]. Together, these findings suggest that functional impairments in type 2 diabetes are not driven by fixed limitations of cristae packing but are better understood within a more complex structural and functional framework.

For the first time in a longitudinal study, we demonstrate that 8 weeks of HIIT increased mitochondrial cristae density by ~7%, irrespective of type 2 diabetes or obesity. This finding contrasts with previous longitudinal human studies. Menshikova et al [50] reported biochemical signatures of cristae expansion without ultrastructural confirmation, while Nielsen et al [23] and Lüthi et al [30] found no changes in cristae density after aerobic or resistance training. This discrepancy with our findings likely reflects both methodological precision and the potency of the HIIT stimulus. By quantifying a minimum of 49 mitochondrial profiles per sample (_est_CE of 0.05), we effectively doubled the resolution compared with earlier studies [23, 31], enabling detection of a modest increase in cristae density (~7%) that may otherwise have remained below the threshold of detection.

An increase in mitochondrial cristae density aligns with cross-sectional studies consistently showing higher cristae densities in endurance- or strength-trained athletes compared with untrained individuals [23, 31–33, 51], with trained muscle approaching ~35 µm^2^ µm^–3^ compared with ~25 µm^2^ µm^–3^ in sedentary counterparts. Seminal animal studies have further demonstrated denser cristae packing following chronic electrical stimulation of muscles in rabbits [52] and cats [53], or after prolonged running in rats [54]. Collectively, these findings reinforce the view that cristae density is a hallmark of endurance adaptation.

A key finding was that adaptations were most pronounced in type 2 fibres and in the intermyofibrillar compartment. This is relevant because type 2 fibres in type 2 diabetes are often characterised by a lower oxidative enzyme activity and higher lipid content compared with lean individuals [55]. Our data show that these fibres, despite their metabolic disadvantage, retain high plasticity when exposed to sufficient exercise stimuli, narrowing the gap with type 1 fibres. At the subcellular level, the greater adaptation in the intermyofibrillar compartment is consistent with its close coupling to contractile activity and substrate utilisation, and aligns with the findings of Buser et al [56], who showed disproportionate remodelling of subsarcolemmal vs intermyofibrillar mitochondria during cold adaptation in rats, underscoring compartment-specific plasticity of cristae organisation. Importantly, we observed that the increase in cristae surface area per muscle volume exceeded the change in mitochondrial volume density, suggesting that exercise training induces not only more mitochondria but also structurally more complex organelles, with potential functional implications for oxidative metabolism. Together, these data highlight that mitochondrial ultrastructure is both adaptable and functionally relevant, with remodelling occurring in a fibre- and compartment-specific manner, and that this adaptive response is preserved in muscles from patients with type 2 diabetes.

Increased mitochondrial cristae density has been proposed to offset the trade-off between mitochondrial volume density and cellular space occupancy [23]. Our data demonstrate that short-term training in non-athletes with low mitochondrial volume density increases cristae density, suggesting that this adaptation can occur independently of spatial constraints. Consistently, prolonged altitude exposure has been reported to decrease cristae density while increasing mitochondrial volume density [57], confirming no fixed relationship between these parameters. High-precision studies are needed to determine how mitochondrial cristae density adapts to different training modalities and metabolic stressors.

Given the localisation of electron transport chain complexes within the cristae, mitochondrial cristae density in muscle is likely an important determinant of oxidative capacity [23]. However, additional aspects of the mitochondrial ultrastructure, beyond the scope of the present study, such as cristae morphology and junction organisation [58], mitochondrial network connectivity [59] and focal mitochondrial damage [60], may also influence mitochondrial function. Because these features were not assessed, they may partly account for the relatively modest correlations observed between cristae measures and whole-body oxidative capacity in the present study. Furthermore, our study included only male participants, which limits the generalisability of these findings to women.

In conclusion, skeletal muscle mitochondrial cristae density does not differ between individuals with type 2 diabetes and glucose-tolerant individuals with obesity, and lean individuals, and the capacity for cristae remodelling in response to exercise training is preserved in type 2 diabetes. Notably, HIIT increased cristae surface area per muscle volume more than mitochondrial volume density alone, reflecting combined structural and volumetric remodelling. These findings highlight the plasticity of mitochondrial architecture and support HIIT as a potent stimulus for improving muscle oxidative potential, even in metabolically compromised skeletal muscle.

## Supplementary Information

Below is the link to the electronic supplementary material.

ESM Figures (PDF 461 KB)

## Data Availability

The datasets generated and/or analysed during the current study are available from the corresponding authors upon reasonable request.

## Abbreviations

_est_CE: Estimated coefficient of error
HIIT: High-intensity interval training
MICOS: Mitochondrial contact site and cristae organising system
OPA1: Optic atrophy 1
RER: Respiratory exchange ratio
TEM: Transmission electron microscopy
