# Supplementary material for: Mitochondrial cristae density is increased following high-intensity interval training in men with type 2 diabetes
Source: Diabetologia. 2026 Mar 8;69(6):1600–17. doi: 10.1007/s00125-026-06694-6 (PMC13109237; doi:10.1007/s00125-026-06694-6)
Supplement: Supplementary file 1 — ESM Figures (PDF 461 KB) [file 125_2026_6694_MOESM1_ESM.pdf]

Electronic supplementary material

ESM Fig. 1 and 2

Mitochondrial cristae density is increased following high-intensity interval training in men with type 2 diabetes

Martin E. de Almeida, Niels Ørtenblad, Amalie B. Platz, Maria H. Petersen, Kurt Højlund and Joachim Nielsen

ESM Fig. 1

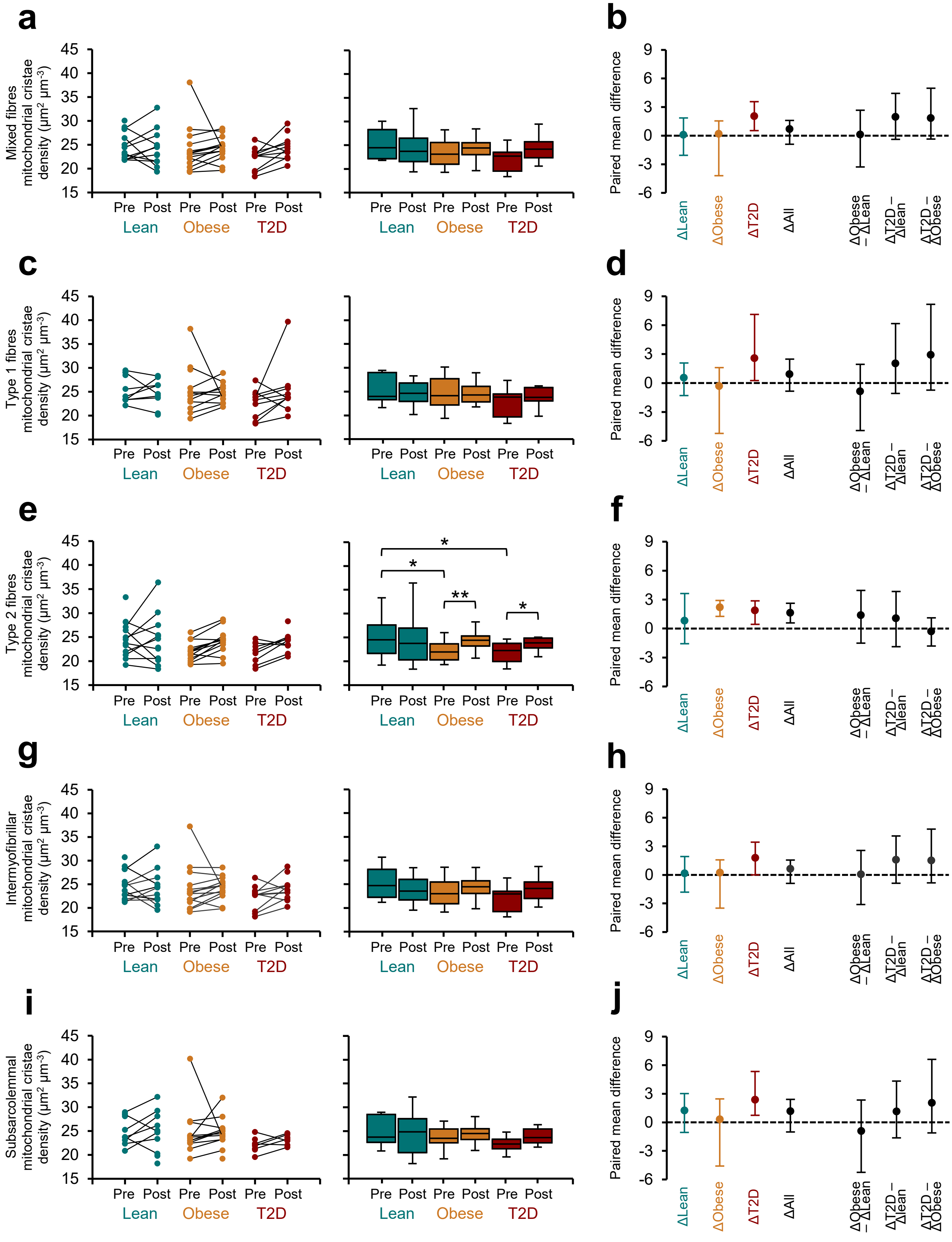

Effect of high-intensity interval training (HIIT) on mitochondrial cristae density ($_{est}$CE <10%) in skeletal muscle fibres and subcellular regions. Cristae density is shown for (a–b) mixed fibres, (c–d) type 1 fibres, (e–f) type 2 fibres, and (g–h) intermyofibrillar and (i–j) subsarcolemmal regions. For each category, the left and middle panels (a, c, e, g, i) present individual pre- and post-HIIT values (slopegraphs) alongside group-wise distributions (boxplots with medians and interquartile ranges). Mixed-effects model results (main effect of group, main effect of time, and the group × time interaction) were: Mixed fibres (group p=0.482; time p=0.162; interaction p=0.064), type 1 fibres (group p=0.411; time p=0.311; interaction p=0.220), type 2 fibres (group p=0.371; time p=0.005; interaction p=0.034), intermyofibrillar (group p=0.462; time p=0.205; interaction p=0.134), and subsarcolemmal (group p=0.634; time p=0.185; interaction p=0.189). Asterisks indicate significant group or time effects: *p<0.05, **p<0.01. The right panels (b, d, f, h, j) display paired mean differences and delta–delta (ΔΔ) comparisons. Colours represent groups: Lean (teal), Obese (orange), and T2D (red). Participants were included if ≥8 mitochondrial profiles were analysed ($_{est}$CE <10%). Individual data and boxplots: Lean: n=11–13 pre, n=10–12 post; Obese: n=13–14 pre, n=12–14 post; T2D: n=10–11 pre, n=9–11 post. Paired mean differences: Lean: n=9–11; Obese: n=11–14; T2D: n=8–10.

ESM Fig. 2

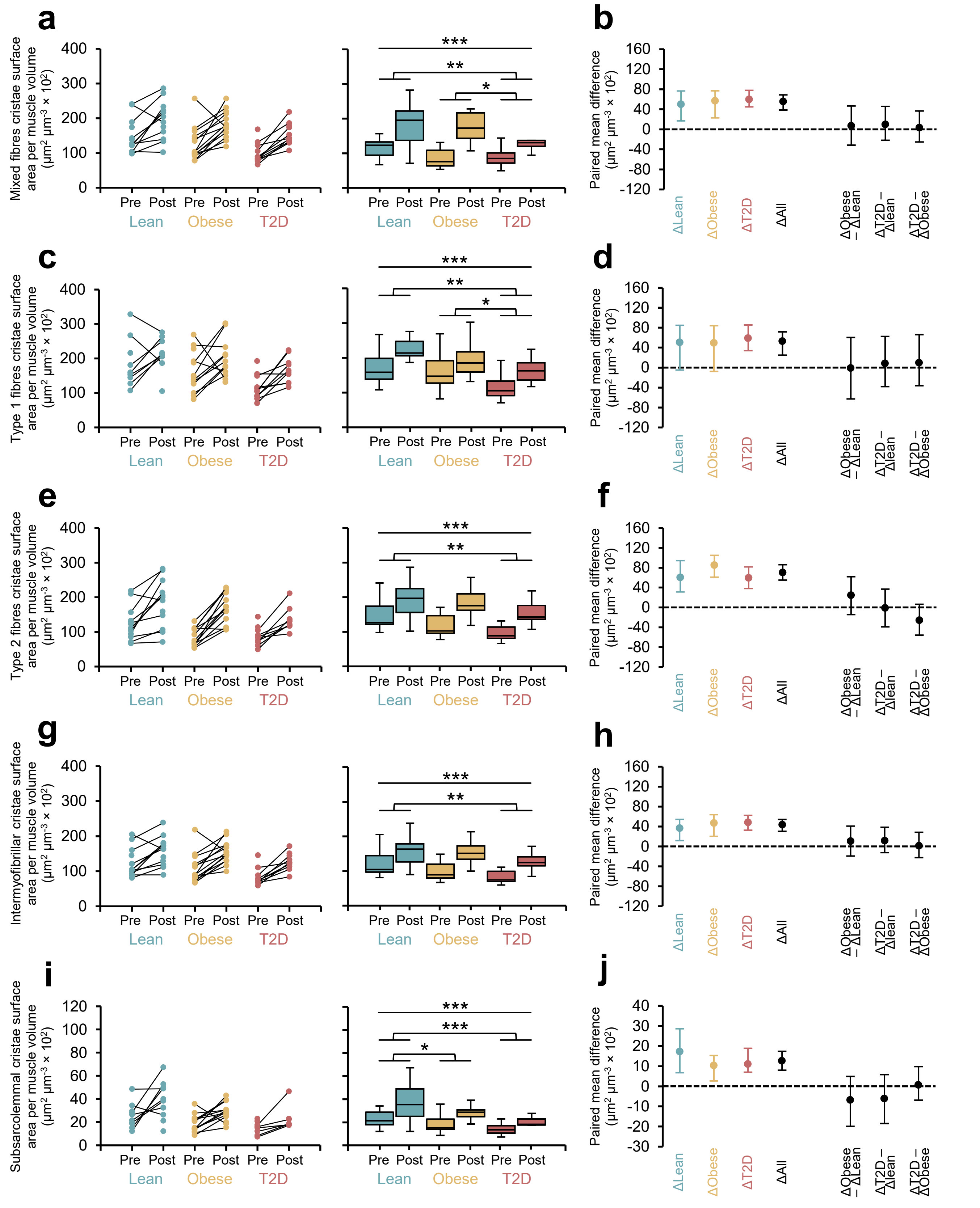

Effect of high-intensity interval training (HIIT) on mitochondrial cristae surface area per muscle volume ($_{est}$CE <10%) in skeletal muscle fibres and subcellular regions. Cristae surface area per muscle volume is shown in mixed fibres (**a**, **b**), type 1 fibres (**c**, **d**) and type 2 fibres (**e**, **f**), and intermyofibrillar (**g**, **h**) and subsarcolemmal regions (**i**, **j**). Panel layout and presentation of individual data, boxplots, paired mean differences, and Δ-Δ comparisons are as for ESM Fig. 1. Mixed-effects model results (main effect of group, main effect of time, and the group × time interaction) were as follows: mixed fibres, group p=0.006, time p<0.0001, interaction p=0.079; type 1 fibres, group p=0.002, time p<0.001, interaction p=0.862; type 2 fibres, group p=0.023, time p<0.0001, interaction p=0.123; intermyofibrillar region, group p=0.020, time p<0.0001, interaction p=0.421; and subsarcolemmal region, group p=0.001, time p<0.0001, interaction p=0.865. *p<0.05, **p<0.01, ***p<0.001. Data from participants were included if ≥8 mitochondrial profiles analysed ($_{est}$CE <10%). Individual data and boxplots: lean, n=11–13 pre, n=10–12 post; obese, n=13–14 pre, n=12–14 post; type 2 diabetes, n=10–11 pre, n=9–11 post. Paired mean differences: lean, n=9–11; obese, n=11–14; type 2 diabetes, n=8–10. T2D, type 2 diabetes
